# Factors associated with antihypertensive treatment intensification and deintensification in older outpatients

**DOI:** 10.1016/j.ijchy.2021.100098

**Published:** 2021-06-23

**Authors:** Carole E. Aubert, Jin-Kyung Ha, Eve A. Kerr, Timothy P. Hofer, Lillian Min

**Affiliations:** a Department of General Internal Medicine, Bern University Hospital, Inselspital, University of Bern, Bern, Switzerland; b Institute of Primary Health Care (BIHAM), University of Bern, Switzerland; c Center for Clinical Management Research, Veterans Affairs Ann Arbor Healthcare System, Ann Arbor, MI, USA; d Institute for Healthcare Policy and Innovation, University of Michigan, Ann Arbor, MI, USA; e Division of Geriatric and Palliative Medicine, Department of Medicine, University of Michigan, Ann Arbor, MI, USA; f Department of Internal Medicine, University of Michigan, Ann Arbor, MI, USA; g VA Ann Arbor Medical Center VA Geriatric Research, Education, and Clinical Center (GRECC), Ann Arbor, MI, USA

**Keywords:** Hypertension, Medication, Treatment, Deintensification, Intensification, Patterns, Elderly, Veterans

## Abstract

**Background:**

New hypertension performance measures encourage more intensive treatment in older adults. Treatment intensification includes starting new medications and increasing the dose of old ones. Medication dose is particularly important to older adults, given their vulnerability to dose-related side effects. We previously validated a standardized measure of beneficial doses tested in hypertension trials, Hypertension Daily Dose (HDD).

**Aim of the study:**

To test whether changes in treatment intensity using HDD was associated with systolic blood pressure (SBP) and patient characteristics.

**Methods:**

Longitudinal study of all Veterans aged ≥65 years with a diagnosis of hypertension. We defined 3 groups of risk: 1) cardiovascular risk; 2) geriatric/frail; 3) low-risk (comparator). Using multinomial regression, we assessed the probability of deintensification, intensification, vs. stable treatment, according to SBP and group.

**Results:**

Among 1,331,111 Veterans, 19.9% had deintensification, and 29.6% intensification. Deintensification decreased, while intensification increased, with SBP. Compared to low-risk patients, cardiovascular risk patients had 1.11 (95% CI 1.10–1.13) times the odds of intensifying, and geriatric/frail patients 1.45 (95%CI 1.43–1.47) times the odds of deintensifying.

**Discussion:**

Patient-level HDD change was consistent with an expected association with cardiovascular risk and geriatric/frail conditions, suggesting that HDD can be used longitudinally to assess hypertension treatment modification in large health systems.

## 1 Introduction

It can be challenging to measure hypertension treatment intensification and deintensification for a primary care population for a number of reasons. First, many health systems do not have ready access to pharmacy records in order to verify what medications are actually dispensed. But even for those that do, observed blood pressure is highly variable and both medications and doses are changed frequently. There are many medication classes and choices within classes, all of which have different potency. Previous studies of community populations where pharmacy records are available have focused solely on medication count or blood pressure (BP) control [1,2]. We desired to more precisely study total dose burden over time, an issue particularly important for older adults, who are more vulnerable to dose-related and polypharmacy-related adverse drug events [3].

In prior work focused on only those patients with aggressive hypertension treatment (systolic blood pressure [SBP] <120 ​mmHg and ≥3 antihypertensive medications), we developed a new measure to assess the intensity of hypertension pharmacological treatment (i.e., total daily dose) on any given day based on pharmacy fill data [4,5]. Unlike previous methods, this new measure uses standardized doses based on trial evidence-based beneficial doses used to treat hypertension. In the present study, we applied this new measure to all 1.3 million older Veterans with hypertension diagnosis who were receiving any level of intensity of hypertension medication treatment, from the Veterans Health Administration (VHA) during 2011–2013. The risk-benefit tradeoff of BP treatment is most likely to be favorable if the degree of treatment intensity is matched to risk of cardiovascular events vs. the risk of side effects. Thus, to assess whether our new measure can be used to track hypertension treatment intensity using health system data, we looked at whether the probability of deintensifying or intensifying was consistent with the expected direction of effect for patients with cardiovascular risk and geriatric/frail conditions using our new measure.

## 2 Methods

### 2.1 Patients and setting

We used administrative and clinical data from all US Veterans Affairs (VA) facilities available through the Clinical Data Warehouse (CDW), including demographics, healthcare encounters, and VA pharmacy fills. CDW data were linked to Medicare Part D pharmacy claims (Department of Veterans Affairs, VA Health Services Research and Development Service, VA Information Resource Center (#02–237 and 98–004)). This research was conducted under Human Subjects review (VA IRB 2015–286).

We included all Veterans aged ≥65 years with a diagnosis of hypertension (International Classification of Diseases [ICD]-9 401.x), established primary care at the VHA (≥2 visits between July 1, 2009 and June 30, 2011), and ≥1 eligible visit during the study period (July 1, 2011 to June 30, 2013). Eligible visits included all outpatient visits from general internal medicine, geriatrics, and specialties managing hypertension (cardiology, endocrinology, nephrology, neurology), during the study period. The first visit during the study period was used as baseline visit.

### 2.2 Outcome

The primary outcome of interest was one of three possible treatment strategies revealed after the baseline visit representing a choice between modifying or keeping the same antihypertensive treatment intensity: 1) stable treatment (no dose change; reference); 2) deintensification (total standardized dose decrease); and 3) intensification (total standardized dose increase). We calculated dose change between baseline visit and 90 days after that visit to determine the treatment strategy. The 90-day time frame was chosen because it corresponds to the usual refill period. Patients who died within 90 days after baseline visit were included only for analyses of the baseline measurements, as they could not be assigned a treatment strategy.

To determine the complete antihypertensive medication regimen, we extracted data from both VA and Medicare Part D fills, and applied a measure that we previously validated in a sample of this Veterans population, which uses prefills (within 186 days before a visit) and refills (within 186 days after) to determine the most likely medication (name, class, dose) that a patient was receiving on any day (i.e., not only visit days) [5]. To allow comparison across all antihypertensive medications, we defined a standardized moderate daily dose (“Hypertension Daily Dose” [HDD]) for each medication, with one HDD corresponding to half the maximum beneficial dose demonstrated in hypertension trials, as published in the Joint National Commission (JNC) 7 and 8 and American Hospital Association/American College of Cardiology (AHA/ACC) guidelines [[6], [7], [8]]. For each patient, the standardized total daily dose was obtained by summing the HDDs of each medication. For example, a patient on lisinopril 20 ​mg (half maximal dose: 1 HDD) and hydrochlorothiazide 50 ​mg (maximal dose: 2 HDDs), was assigned 3 HDDs.

### 2.3 Measures

We defined baseline BP as the mean of BP measured during baseline visit and during the last visit before baseline to increase the precision of the baseline measurement. We classified patients into three groups (**Supplemental Text 1**). “Cardiovascular risk” comorbidity grouping, included patients with conditions increasing the likelihood to benefit of intensive antihypertensive treatment (diabetes mellitus, cardiovascular disease, congestive heart failure, chronic kidney disease, smoking, hyperlipidemia), yet they were free of geriatric/frailty criteria described under group 2. “Geriatric/frail” comorbidity grouping, included patients with dementia, psychotic disorder, fall risk, osteoporosis, skin ulcer, and/or nutritional deficiency. “Healthy/low-risk” grouping, were patients not included in the previous groups, and was used as reference. The three groups were mutually exclusive and defined based on ICD-9 codes (**Supplemental Text 1** and Supplemental Table 1**).**

### 2.4 Statistical analyses

We described the distribution of HDD and medication count according to baseline systolic BP (SBP) and diastolic BP (DBP), respectively. We assessed the incidence of each treatment strategy (i.e., deintensification, intensification, stable treatment). We used a multinomial logistic regression analysis to assess the probability of deintensification, intensification, and stable treatment, adjusted for age, baseline standardized antihypertensive medication dose, baseline SBP, and comorbidity variables not included in the 3 groups above (Supplemental Text S2). We assessed separately the 3 group risks (cardiovascular, geriatric/frail, healthy/low-risk). We performed all analyses using Stata 15.1 (Stata-Corp LP, College Station, TX, USA), and SAS Enterprise Guide 7.1 (SAS Institute).

## 3 Results

### 3.1 Baseline characteristics

The sample included 1,331,111 patients, with a mean age of 76.1 (SD 7.5) years, 98.2% males, and a mean SBP of 129.8 (SD 12.8) mmHg (Table 1). Most patients (N ​= ​943,341; 70.9%) had multimorbidity, with a mean of 3.5 (SD 3.6) chronic conditions in addition to hypertension. One-third (32.2%) had cardiovascular disease, 28.7% had diabetes mellitus, and 11.0% had atrial fibrillation or flutter. With respect to the comorbidity groupings, 31.4% of the patients were in the cardiovascular risk group, 29.0% in the geriatric/frail group, and 39.6% in the healthy/low-risk group.

**Table 1:** Baseline characteristics of the patients (N = 1,331,111).

Characteristic	N (%) or mean (SD)
Age, years	76.1 (7.5)
Baseline systolic blood pressure, mmHg	129.8 (12.8)
<120.0	279,390 (21.0)
120.5–140.0	829,344 (62.3)
>140.0	222,377 (16.7)
Baseline Hypertension Daily Dose	1.9 (1.9)
*Chronic conditions*	
- N of conditions in addition to hypertension	3.5 (3.6)
- Multimorbidity (≥2 chronic conditions)	943,341 (70.9)
- Diabetes mellitus	382,419 (28.7)
- Current smoking	124,241 (9.3)
- Cardiovascular disease^a^	428,649 (32.2)
- Atrial fibrillation or flutter	146,209 (11.0)
- Chronic kidney disease	156,394 (11.8)
- Cancer	169,514 (12.7)
*Subgroup*^b^	
- Cardiovascular risk	430,455 (32.3)
- Geriatric/frail	88,137 (6.6)
- Healthy/low-risk	812,519 (61.0)

Abbreviations: N, number; SD, standard deviation.

a Cardiac, peripheral, and/or cerebral vascular disorder.

b Details of definition in Supplemental Text 1.

#### 3.1.1 Blood pressure control and antihypertensive medication (Fig. 1)

The SBP was <140 ​mmHg in 1,108,734 patients (83.3%), including 279,390 (25.2%) with an SBP <120 ​mmHg, while the DBP was <90 ​mmHg in 97.6% (N ​= ​1,298,662) of the patients. Mean standardized medication dose increased with SBP, with a mean HDD of 1.8 (SD 1.8) in patients with an SBP <120 ​mmHg, 1.9 (SD 1.9) in those with an SBP 120–140 ​mmHg, and 2.2 (SD 2.2) in those with an SBP >140 ​mmHg (p ​< ​0.001). Overall, 19.1% (N ​= ​253,648) of patients had no antihypertensive medication at baseline, a proportion that was similar across all SBP categories.

#### 3.1.2 Incidence of deintensification and intensification

Within 90 days of baseline visit, 13,415 (1.0%) patients died and could thus not be assigned a treatment strategy. Among the remaining patients, 262,238 (19.9%) had deintensification, and 389,351 (29.6%) had intensification of their antihypertensive treatment. 59.3% of patients with deintensification had only a total dose decrease, and 63.2% of those with intensification had only a total dose increase, without changing the total number of medications, while the remaining 40% had changes in the number of medications.

#### 3.1.3 Factors associated with deintensification and intensification (Fig. 2)

Deintensification was more likely at lower SBPs, with an adjusted probability (95%CI) of 23.8% (23.6–24.0%) at SBP ​= ​100 ​mmHg, and 15.8% (15.6–15.9%) at SBP ​= ​160 ​mmHg. On the other hand, intensification occurred with an absolute probability of 20.2% (20.1–20.4%) at SBP ​= ​100 ​mmHg, and 40.7% (40.4–40.9%) at SBP ​= ​160 ​mmHg. Consistent with expectation, geriatric/frail patients were treated more conservatively, with a higher probability of deintensification, particularly when SBP was low (Fig. 2). They had 1.45 (95%CI 1.43–1.47) times the odds of deintensification compared to the healthy/low-risk group. Also consistent with expectation, patients at higher cardiovascular risk were more likely to intensify than healthy patients, with an OR of 1.11 (95%CI 1.10–1.12) (Fig. 2). The healthy/low-risk patients were similar to cardiovascular risk patients in their likelihood to intensify when SBP was above 140 ​mmHg.

**Fig. 1:**
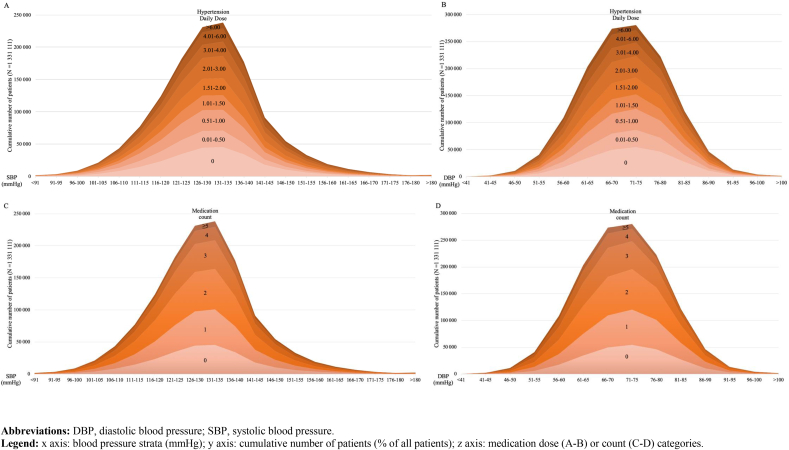
Distribution of baseline antihypertensive medication regimen, according to baseline blood pressure in all patients (N ​= ​1,331,111): A) dose and SBP; B) dose and DBP; C) medication count and SBP; D) medication count and DBP.

**Fig. 2:**
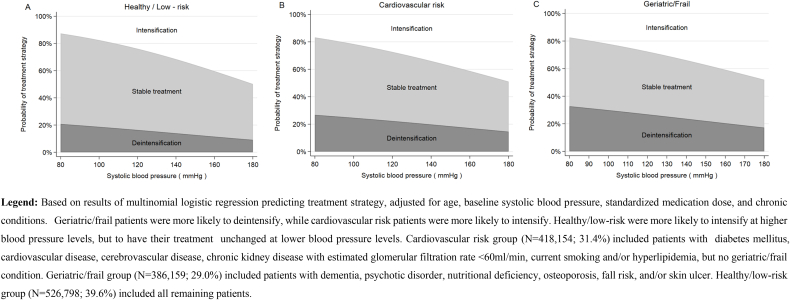
Probability of each treatment strategy according to baseline systolic blood pressure in three subgroups based on comorbidities.

## 4 Discussion

In this large-scale nationwide study using nearly complete pharmacy data sources within the largest US healthcare system, we described the longitudinal modification in hypertension treatment intensity over time in more than 1.3 million older Veterans, using a new more comprehensive measure of treatment intensity based on demonstrated beneficial doses in hypertension trials. At three months, 20% and 30% of patients had received deintensification or intensification of their antihypertensive medication regimen, respectively. Geriatric/frail patients were treated more conservatively, with a higher probability of deintensification, while patients at higher cardiovascular risk were more likely to intensify. Healthy/low-risk patients were more likely to deintensify at low SBP, but to intensify at higher SBP.

At baseline in 2011–2013, 82% of the patients had a BP controlled to <140/90 ​mmHg, corresponding to the treatment goal at that time [9]. This result is consistent with often-cited report that 76% of Veterans had a SBP <140/90 ​mmHg in 2010, a yearly improvement in control rates of 3%, and a level of control better than the rest of the nation [10]. On average, each older Veteran was receiving two medications at half the maximal beneficial dose, a dose similar to the SPRINT control group's mean medication count [11].

Using our new measure to capture treatment intensity modification over time, we observed an expected inverse relationship between SBP and deintensification, and a positive association of SBP with intensification. We also encouragingly found a relationship between baseline SBP and change in treatment intensity that varied according to patient comorbidities and cardiovascular risk. Geriatric and frail patients were more likely to have their treatment deintensified than healthier patients or those at high cardiovascular risk. About 20% of patients at low cardiovascular risk (group 3) had deintensification when SBP was low (<100 ​mmHg). Clinically, we would expect that providers would be more likely to deintensify medication for this low-risk group because they understood that they would have less benefit from tight BP control. However, they were just as likely as the cardiovascular risk patients to be escalated in care if indicated by high SBP. The expected relationship that we observed between SBP, comorbidities and treatment modification, suggests that our measure is a reliable way to assessing antihypertensive medication intensity in this patient population.

Our measure assesses both the number and total standardized dose of antihypertensive medications using administrative data available through the VA electronic medical record and pharmacy database. This measure can be adapted to other health systems that can get access to both observed BP and prescription fills, with several implications. First, total dose burden could supplement traditional measurement of hypertension control by BP values and antihypertensive medication count. The continuous measure of medication treatment intensity can track more closely than medication counts any changes in the intensity of hypertension treatment. Second, it could be used with observational data to assess the outcomes of deintensification and intensification of antihypertensive medication in patients who were excluded from hypertension trials. Finally, it could be used to evaluate the patterns of hypertension treatment (e.g., several medications at low dose or fewer medications at higher dose) over time and in response to new evidence and quality improvement efforts.

### 4.1 Strengths and limitations

We discuss several limitations. First, this study of older Veterans was predominantly males, so that the results may not be generalizable to females. Second, because hypertension control has been found to be better in Veterans than in the general population (76% vs 50% with BP ​< ​140/90 ​mmHg) [10], it is possible that the predictors of deintensification and intensification may differ from the general older US population. Third, pharmacy records do not contain an explicit indication for changes of antihypertensive medication intensity. We could not account for instances where the treatment may have been modified for a non-hypertension condition (e.g., arrhythmia control or neurohormonal modulation in heart failure)*,* potentially resulting in seemingly paradoxical clinical actions, e.g, patients with low SBP receiving an “intensification” or patients with high SBP receiving a “deintensification”. Fourth, some patients with high BP may have appeared to be deintensified but were actually non-adherent resulting in a decrease in HDDs*.* Finally, pharmacy fills do not directly measure actual day-to-day variation in medication consumption or lack of adherence to prescribed and filled regimens. Our measure is able to infer the likely decrease in consumption when patients delay their refills or cease to refill them [12,13], and other studies have demonstrated that pharmacy fills are a reliable method to assess medication consumption [14,15].

This study has several strengths as well. First, we had access to a near-universal medication data source (both VA and Medicare pharmacy fills) in a very large national sample of patients across all US regions being cared for in the largest US healthcare system. Second, we used a measure that assesses both medication count and standardized dose that we validated in a sample of this Veterans population [12,13], and we were able to study the longitudinal patterns of treatment. Third, we averaged two measurements of SBP to define baseline SBP, thus reducing measurement error in a key covariate. Finally, we used objective data (including ICD-9 codes and laboratory data, and accounting for date of diagnosis) to categorize the patients into three subgroups of risk related to the probability to deintensify or intensify hypertension treatment.

## 5 Conclusion

This study provides the first longitudinal insight into specific patterns of hypertension treatment intensity among 1.3 million older Veterans across a national healthcare system with respect to comorbidities and cardiovascular risk. In addition, changes in HDD were consistent with an expected direction of effect with cardiovascular risk and geriatric/frail conditions, thus suggesting that HDD can be used longitudinally to assess hypertension treatment modification for patients in large health systems.

## Disclosures

The authors have nothing to disclose.

## Funding

This research was funded by R01 from the 10.13039/100000049National Institute on Aging (Min AG047178) and the Veterans Health Administration (Min IIR 14–083). Dr. Aubert was supported by an Early Postdoc.Mobility grant from the 10.13039/100000001Swiss National Science Foundation (grant P2LAP3_184042). The funders had no roles in design and conduct of the study; collection, management, analysis, and interpretation of the data; preparation, review, or approval of the manuscript; and decision to submit the manuscript for publication.

## Credit author statement

**Carole E. Aubert:** Conceptualization, Methodology, Formal analysis, Writing – original draft preparation. **Jin-Kyung Ha:** Formal analysis, Writing-Reviewing. **Timothy P. Hofer:** Methodology, Writing-Reviewing and Editing. **Eve A. Kerr:** Methodology, Writing-Reviewing and Editing. **Lillian Min:** Conceptualization, Methodology, Funding acquisition, Supervision, Writing-Reviewing.

## Authors contribution

Dr Aubert had full access to all of the data in the study and takes responsibility for the integrity of the data and accuracy of the analyses. Study concept and design: Aubert, Hofer, Kerr, Min. Acquisition and analysis of data: Aubert, Ha, Min. Interpretation of data: Aubert, Hofer, Kerr, Min. Drafting of the manuscript: Aubert. Critical revision of the manuscript for important intellectual content: All authors.
