# Supplementary material for: Factors associated with antihypertensive treatment intensification and deintensification in older outpatients
Source: Int J Cardiol Hypertens. 2021 Jun 23;9:100098. doi: 10.1016/j.ijchy.2021.100098 (PMC8254109; doi:10.1016/j.ijchy.2021.100098)
Supplement: Multimedia component 1 [file mmc1.docx]

**Supplemental Text 1.** Definition of subpopulations.

We defined three subgroups of patients, according to the likeliness to benefit from more or less intensive hypertension treatment, based on cardiovascular risk and geriatric/frailty conditions:

**Group 1: “cardiovascular risk” group (≥1 criterium, and no criterium of group 2):**

- diabetes mellitus

- cardiovascular disease

- cerebrovascular disease

- chronic kidney disease with estimated glomerular filtration rate <60ml/min

- current smoking

- hyperlipidemia

**Group 2: “geriatric/frail” group (≥1 criterium):**

- dementia

- psychotic disorder

- nutritional deficiency

- osteoporosis

- fall risk

- skin ulcer

**Group 3: “healthy/low-risk” group (comparator group):**

- none criterium of group 1 or group 2

**Supplemental Text 2.** List of comorbidities not included to classify the patients in the 3 groups above.

Lung disease, gastro-intestinal/liver disease, overweight, atrial fibrillation/flutter, defecation disorder, urinary disorder, osteoarticular disorder, cancer, sleep/mood disorder, substance abuse.

**Supplemental Table 1.** Definition of chronic conditions.

| **Conditions** | **International Classification of Diseases-9 codes** |
| --- | --- |
| ***General conditions*** | |
| Anemia | 280.0-285.9 |
| Chronic kidney disease | 249.4X, 250.4X, 271.4, 274.10, 403.XX, 404.XX, 572.4, 582.XX, 583.6, 585-590.01 |
| Lung disorder | 491.0-505, 506.4, 508.1, 512.83, 515, 516.XX, 517.2, 518.83, 518.84 |
| Malignancy | 140.0-172.9, 174.0-209.79, 789.51 |
| Psychiatric or sleep disorder | 290.13, 290.21, 290.43, 291.82, 292.84, 292.85, 293.83, 293.84, 296.XX, 300.01, 300.02, 300.4, 307.4X, 309.1, 311.XX, 327.XX, 780.5X, V69.4 ; 290.12, 290.20, 290.42, 290.8, 290.9, 291.XX, 295.XX, 297.XX, 298.XX ; 291.0-292.9, 303.00-305.93 |
| Peptic or liver disorder | 070.2-070.33, 070.44, 070.54, 070.7X, 456.0X, 456.1X, 530.0-530.6, 530.85, 531.40-531.91, 532.40-532.91, 533.4X, 536.3, 571.0-573.9, 787.2X |
| ***Heart cluster*** | |
| Arrhythmia | 427.31, 427.32, 427.81 |
| Heart failure / valve disorder | 394.0-398.99, 402.XX, 404.XX, 416.0-416.9, 424.XX, 425.XX, 428.XX, 429.3, 429.4, 429.81-429.89, 746.XX, V42.1, V42.2, V43.2-V43.22, V43.3 |
| ***Cardiovascular conditions*** | |
| Cardiac or peripheral vascular disorder | 410.0-414.9, 427.5, 429.2, 440.0-442.9, 443.1-445.89, 557.0, 557.1, 557.9, 996.03, V45.81, V45.82 |
| Cerebrovascular disorder | 433.0-438.9, 997.02, V12.54 |
| Diabetes mellitus | 249.XX, 250.XX, 357.2, 362.01-362.07, V58.67 |
| Obesity or overweight | 278.0X, V85.30-V85.45 |
| Current smoking | NA* |
| Hyperlipidemia | NA* |
| ***Geriatric conditions*** | |
| Arthritis or joint pain | 712.XX, 714.XX, 715.XX, 719.4X, 721.0-721.3, 721.90, 721.91, 724.1, 724.2, 724.5 |
| Cognitive disorder | 290.XX, 292.8X, 294.XX, 330.XX, 331.XX, 438.0, 780.93, 780.97, 797.XX, 799.59, V40.31 |
| Defecation disorder | 560.32, 560.39, 564.0X, 564.1, 564.5, 787.6X |
| Fall risk | 340-342.91, 356.XX, 357.XX, 386.XX, 438.2-438.22, 438.40-438.42, 438.84, 438.85, 458.0, 719.7, 728.87, 780.2, 780.4, 781.1, 781.2, 781.3, V15.88 |
| Hearing impairment | 388-389.9X, V41.2, V53.2 |
| Nutrition deficiency | 260-269.9, 783.0, 783.2-783.3, 799.4, V85.0 |
| Osteoporosis | 733.00-733.19, 733.93-733.98, V56.68 |
| Severe vision impairment | 360.21, 360.41, 360.42, 365.73, 369.XX |
| Skin ulceration | 707.XX, 440.23, 440.24, 454.0, 454.2, 459.11, 459.13 |
| Urination and prostate disorder | 788.2X, 788.20, 788.21, 788.29, 788.3X, 788.6, 788.61-788.65, 788.69, 788.8, 788.9, 788.91, 788.92, 595.1, 595.2, 596.0, 596.4, 596.5, 596-596.55, 596.59, 600.XX, 601.1X |

* Not defined using International Classification of Diseases-9 codes. Current smoking was defined retrieving information from the Corporate Data Warehouse and Medicare diagnosis files. Hyperlipidemia was defined using laboratory data, as cholesterol LDL >1.60 mg/dl, or total cholesterol/HDL cholesterol ratio >4.
